# The Mechanism of miR-141 Regulating the Proliferation and Metastasis of Liver Cancer Cells by Targeting STAT4

**DOI:** 10.1155/2021/5425491

**Published:** 2021-10-12

**Authors:** Lili Ma, Hui Shao, Huazhong Chen, Qilong Deng

**Affiliations:** ^1^Hepatology and Infectious Diseases Center, Taizhou Hospital of Zhejiang Province Affiliated to Wenzhou Medical University, Linhai 317000, Zhejiang, China; ^2^Rehabilitation Medical Center, Taizhou Hospital of Zhejiang Province Affiliated to Wenzhou Medical University, Linhai 317000, Zhejiang, China

## Abstract

**Background:**

In recent years, it has been reported that miRNA can be used as one of the markers of tumor diagnosis, treatment, and prognosis (including liver cancer), and it plays an important role in tumorigenesis. However, there are still very few studies on the mechanism and role of miR-141 in liver cancer.

**Methods:**

qRT-PCR was used to test the expressions of miR-141 and STAT4 in collected liver cancer tissues and adjacent tissues, cultured liver cancer cell lines MHCC97H, Hep3B, and Huh7, and normal human liver cells HL7702. After processing the results of the qRT-PCR experiment, liver cancer cell MHCC97H which has the lowest expression level was decided to be taken as the research object. miR-NC, miR-141 mimics, si-NC, si-STAT4, miR-141 mimics and pcDNA-NC, and miR-141 mimics and pcDNA-STAT4 were transfected into MHCC97H cells, respectively. The MTT assay was used to detect the proliferation of each group of cells, and the Transwell test was used to detect the effect of miR-141 on cell proliferation, migration, and invasion. The interaction between miR-141 and STAT4 was verified by the dual-luciferase reporter experiment, and the expression level of Cyclin D1 and MMP2 was detected by the western blot.

**Results:**

Compared with normal cell HL7702, the expression level of miR-141 in liver cancer cell lines was relatively low (*P* < 0.05) and the expression level of STAT4 in liver cancer cell lines was relatively high (*P* < 0.05) after testing the expression level of STAT4; transfecting miR-141 mimics or Si-SLBP can inhibit cell proliferation, migration, and invasion; dual-luciferase reporter experiments confirmed that miR-141 can specifically bind to the 3′UTR of STAT4; cotransfection of miR-141 mimics and pcDNA-STAT4 can antagonize the effects of miR-141 mimics on cell proliferation, migration, and invasion.

**Conclusion:**

miR-141 can target the STAT4 gene expression to inhibit the proliferation, migration, and invasion of liver cancer cells.

## 1. Introduction

Liver cancer, colorectal cancer, gastric cancer, and lung cancer are the four cancers with the highest mortality rate in the world, so scientists are doing more and more research on liver cancer [1]. Hepatocellular carcinoma (HCC) is the main subtype of primary hepatic carcinoma, accounting for more than 80% of primary hepatic carcinoma [2]. Studies have shown that a variety of risk factors can induce the occurrence of HCC, for example, chronic viral hepatitis B (HBV) or hepatitis C (HCV) infection, which account for more than 80% of global HCC cases [3]. Currently, alcoholic liver disease (ALD) is the most important cause for HCC in the United States and Europe, while in most other developed countries, the number of nonalcoholic fatty liver disease (NAFLD) patients has increased year by year, making NAFLD the most important cause for HCC [4, 5]. With the development of science and technology, the research and treatment of liver cancer have also achieved leapfrog development, but the prognosis of HCC patients is still poor. For example, in Asia, the 5-year survival rate of HCC patients is only 15.38%. This phenomenon occurs for many reasons, such as late treatment, complicated symptoms, and failure of chemotherapy [6–8]. Therefore, how to improve the prognosis of liver cancer patients has become the main reason for exploring the mechanism of liver cancer.

Noncoding RNA refers to those sequences that do not code for proteins or peptides, and it has two types. One is a small noncoding RNA with a length of about 20 to 30 bp. The most common ones are microRNAs (miRNAs), small interfering RNA (siRNA), and piwi-interaction RNA (piRNA). The other type is long noncoding RNA, which is about several hundred bp in length. In cancer research, miRNAs are mostly involved. miRNAs are endogenous noncoding small RNAs, which are mainly used to regulate gene expression by degrading messenger RNA or combining to the three untranslated regions (UTR) of target mRNA. Scientists found that, in primary liver cancer, most of its tumor promoters and suppressors are miRNAs, which indirectly affect cell proliferation, migration, invasion, and development [9, 10]. miRNA is believed to be capable of inhibiting hundreds of target genes after transcription, so they are powerful regulators of gene expression [11, 12].

miRNA regulates about 30% of human genes through the above-mentioned pathways, many of which regulate various carcinogenic molecules and pathways may be located in unstable sites [13, 14]. In most cancers, miRNAs have been proven to be dysregulated. Oncogenic miRNAs (OncomiRs) and tumor suppressor miRNAs are related to carcinogenesis and malignant transformation and contribute to or inhibit the cancer phenotype [15]. Overexpression of OncomiRs has been observed in various tumors. The disorders of miRNAs in cancer are often described as gastrointestinal [16, 17], urinary system [18, 19], gynecology [20], and lung cancer [21]. Recently, more and more reports have described the application of miRNAs as biomarkers or therapeutic targets for HCC [22–24].

In this study, hepatocellular carcinoma cells were taken as the research objects, and the expression of miRNA-141 in hepatocellular carcinoma cells and the effect of targeting STAT4 on the proliferation, migration, and invasion of hepatocellular carcinoma cells were detected using qRT-PCR, MTT, and Transwell compartment method. The possible molecular mechanisms were further explored, to provide potential biomarkers and therapeutic targets for the clinical diagnosis and treatment of liver cancer.

## 2. Materials and Methods

### 2.1. Materials

#### 2.1.1. Clinical Tissue Samples

The liver cancer tissues and adjacent tissues involved in this study were all from the Affiliated Hospital of Harbin Medical University, where a total of 121 patients underwent hepatocellular carcinoma surgery from March 2018 to May 2020. The acquisition of all samples and follow-up related experimental studies were all in line with the requirements of the ethical review committee of our hospital and have gained the informed consent of patients or their family members. After the samples were isolated, they were all embedded and stored in liquid nitrogen by relevant researchers to prepare for the subsequent detection of miR-141 expression.

#### 2.1.2. Cells

The liver cancer cell lines MHCC97H, Hep3B, and Huh7 used in this study and normal human liver cells HL7702 were purchased from the Chinese Academy of Sciences (Shanghai).

#### 2.1.3. Reagents

Fetal bovine serum and DMEM medium were purchased from GIBCO, USA; trypsin-EDTA digestion solution, MTT, and DMSO were purchased from Sigma, USA; SP kit, RIPA lysate, and BCA protein concentration kit were purchased from Beyotime Research Institute; antibodies and protease inhibitors were purchased from Proteintech.

### 2.2. Methods

#### 2.2.1. Cell Culture

In this study, all cell lines, after resuscitation, were cultured in DMEM medium (with double antibody) (GIBCO, Grand Island, NY, USA) containing 10% fetal bovine serum and 1% penicillin/streptomycin, and the cells were cultured in an incubator, with a culture condition of 37°C, 5% CO_2_.

#### 2.2.2. Cell Transfection

MHCC97H, Hep3B, and Huh7 liver cancer cells in the logarithmic growth phase were selected and inoculated into a 6-well plate according to the amount of 1 × 10^6^/well. 2 ML culture medium was added to each well, and transfection was performed when the cell density reached 80%. In this study, Lipofectamine 3000 reagent (Invitrogen, Carlabad, CA, USA) was used in transfection, just following the instructions. First, transfect miR-NC (negative control) and miR-141 mimics group (experimental group) into MHCC97H cells, then transfect Si-NC (negative control) and Si-STAT4 (experimental group) into MHCC97H cells, and finally transfect miR-141 + pcDNA-NC group (transfected with miR-141 mimics and pcDNA-NC) and miR-141 + pcDNA-STAT4 group (transfected with miR-141 mimics and pcDNA-STAT4) into MHCC97H cells, and placed them in an incubator (37°C, 5% CO_2_). After 48 hours, collect these cells for subsequent experiments.

#### 2.2.3. qRT-PCR

qRT-PCR was used to detect the expression level of miR-141 and STAT4 in the liver cancer tissues and adjacent tissues collected. TRIzol (Invitrogen, Thermo Fisher Scientific) was used to extract total RNA. mRNA was reverse transcribed using the reverse transcription kit (TaKaRa), and miRNA reverse transcription was synthesized using the mi Script reverse transcription kit (Qiagen). All operations were carried out in accordance with the instructions on the kit. LightCycler 480 (Roche) fluorescent quantitative PCR instrument was used to detect gene expression, and the reaction conditions were set according to the operating instructions on the fluorescent quantitative PCR kit (SYBR Green Mix, Roche). The thermal cycle parameters were 95°C for 10 s, then 95°C for 5s, 60°C for 10 s, and 72°C for 10 s, with a total of 45 cycles; the last 72°C was extended for 5 min. Quantitative PCR was set to 3 replicates for each reaction. U6 was used for miRNA internal reference, and GAPDH was used for mRNA internal reference. 2-ΔΔCt method was used for data analysis (ΔΔCt = experimental group (Ct target gene-Ct internal reference), control group (Ct target gene-Ct internal reference)), and relative quantitative method was used to calculate the mRNA expression level of each group.

#### 2.2.4. MTT Assay

After the MHCC97H cells were digested with trypsin, they were plated into 96-well plates according to the amount of 4 × 10^3^/well and divided into 3 groups. After culturing for 24, 48, and 72 hours, add 20 *μ*L of MTT solution (concentration of 5 mg/well) to each well, then put the processed 96-well plate in a 37°C incubator for 4 hours. Four hours later, take out the 96-well plate and use a pipette to discard the culture solution. Then, add 150 *μ*L of DMSO to the 96-well plate and shake for 10 min on a shaker. Put the 96-well cell culture plate into the microplate reader, measure the absorbance value of the solution at a wavelength of 490 nm, and save the experimental data at this time. Use GraphPad Prism 5 software to process the experimental data, set the absorbance value as the ordinate and time as the abscissa, and gain the cell proliferation curve through analysis.

#### 2.2.5. Transwell Experiment

A Transwell experiment was used to detect the migration and invasion of MHCC97H cells. Count the cells after going through digestion, centrifugation, and resuspension. Dilute the cells to an appropriate concentration with a cell culture medium without 10% FBS, generally 2.5 × 10^5^ cells/mL. Then, take 200 *μ*L of the diluted cell suspension (without serum) and add it to each cell compartment to make a cell density of 5 × 10^4^. Repeat three times for the experimental group and the control group, respectively, and add 700 *μ*L of cell culture medium containing 15% FBS to the lower layer of the cell compartments. Pay attention to operate gently, to prevent the formation of air bubbles in the lower layer of the cell compartments. Then, place the cell compartments in a 37°C, 5% CO_2_ cell incubator for culture. After culturing for 24 hours, take out the cell compartments for the cell migration experiment. The cell perforation time was longer in the cell invasion experiment, taking out the cell compartments after culturing for 48 hours. Take out the cell compartments at the specified time, wipe the excess cells with a cotton swab, wash with 1 × PBS, fix them with 4% paraformaldehyde for 30 minutes, stain them with crystal violet for 30 minutes, rinse gently with running water, observe, and take pictures under a microscope. Count the number of migrated cells and save the data. In the cell invasion experiment, 100 *μ*L Matrigel glue, and 500 *μ*L serum-free medium were thoroughly mixed. 50 *μ*L of the mixed solution was sucked into the cell compartments and incubated in the incubator for 4h. The rest of the steps were the same as above.

#### 2.2.6. Dual-Luciferase Reporter Experiment

Dual-luciferase reporter gene detecting the target gene of miR-141: construct wild-type vector WT-STAT4 and mutant vector MUT-STAT4, respectively, cotransfect WT-STAT4 and MUT-STAT4 with miR-NC and miR-141 mimics in MHCC97H cells, and detect the relative luciferase activity of cells 24 h after transfection. Transfect miR-NC, miR-141, anti-miR-NC, and anti-miR-141 into MHCC97H cells, and use Western blotting to determine the STAT4 protein expression.

#### 2.2.7. Western Blotting Experiment

Spread the experimental cells evenly into a 6-well plate, culture overnight and then transfect the cells, discard the culture medium after 48 hours of transfection, wash 2-3 times with 1 × PBS, scrape the cells out into a 1.5 mL centrifuge tube with a cell brush, and place on ice. After centrifugation, discard the supernatant, add RIPA lysate to the deposits, sonicate the cells, and repeat 2-3 times to ensure that the protein can be fully lysed. After the sonication, centrifuge for 15 minutes and aspirate the supernatant to obtain the protein sample. Then, use the BCA kit purchased from Beyotime to measure the concentration of the obtained protein. According to the sample size, take the corresponding sample volume and equal volume of 1 × loading, and boil it in boiling water for 5 minutes to denature the protein. Added the sample to the 12.5% SDS-PAGE gel well prepared in advance, put on the electrophoresis cover, set the electrophoresis instrument to 80 V for 30 minutes, and switch to 100 V for 1 hour after the protein sample band entered the separation gel. After the protein band ran away completely, cut the gel and got ready for transfer. The transfer needed to be carried out in an ice bath, with the transfer current constant at 220 mA, for 60 min. After the transfer is completed, put the membrane in the preprepared 5% milk medium and seal it for one hour. After completion, put the membrane into 1 × TBST washing solution to rinse 3 times, each time for 10 minutes. Check the antibody instructions, dilute the antibody proportionally with TBS buffer, place the membrane in the diluted antibody solution, and incubate overnight in a refrigerator at 4°C. The next day, wash the membrane 3 times with 1 × TBST washing solution, each time for 10 min. Transfer to the secondary antibody (horseradish peroxidase-labeled goat anti-rabbit IgG, 1:5000, CoWin Biosciences), incubate on a shaker for 1-2 hours, after the secondary antibody incubation, wash with 1 × TBST solution for 3 times, each time for 10 min. Finally, do coloration. According to the size of the PVDF membrane, drop an appropriate volume of ECL luminescent liquid evenly on the membrane, and the gel imager was used for exposure. Collect the images through Image studio software; adjust the strength of the target protein band after the exposure; label the experiment date, name, and protein sample loading sequence; and save the picture.

### 2.3. Statistical Processing

Statistical software SPSS21.0 was used to analyze the data. The measurement data were expressed as *χ* ± *s* and all accorded with the normal distribution. The comparison between the two groups was performed by independent sample *t*-test, and the comparison between multiple groups was performed by one-way analysis of variance. When *P* < 0.05, the difference was statistically significant.

## 3. Results

### 3.1. The Expression Levels of miR-141 and STAT4 in Liver Cancer Tissues and Cell Lines

**Figure 1:**
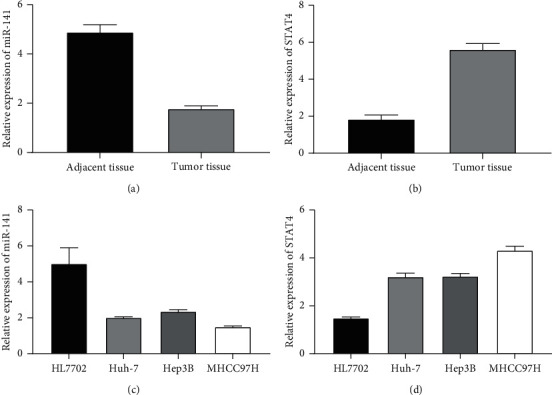
Expression levels of miR-141 and STAT4 in liver cancer tissues and cell lines. (a) Expression of miR-141 in liver cancer tissues. (b) Expression of STAT4 in liver cancer tissues. (c) Expression of miR-141 in HCC cell lines. (d) STAT4 expression in HCC cell lines. Note: *P* < 0.05.

First, qRT-PCR was used to detect the expression levels of miR-141 and STAT4 in the cancer tissues and normal liver tissues of the 121 liver cancer patients. The results showed that, compared with normal tissues, the expression levels of miR-141 in liver cancer tissues were significantly downregulated as in Figure 1(a), and the expression levels of STAT4 in liver cancer tissues were significantly upregulated as in Figure 1(b). At the same time, the expression levels of miR-141 in MHCC97H, Hep3B, and Huh7 liver cancer cells were also significantly downregulated compared with normal cells HL7702 as in Figure 1(c). The expression levels of STAT4 were higher in hepatocellular carcinoma cell lines than in normal HL7702 cells as in Figure 1(d). Because MHCC97H cells had a lower expression level of miR-141 compared with other hepatocellular carcinoma cells, this paper selected MHCC97H cells for a follow-up study.

### 3.2. Effect of miR-141 Overexpression on Cell Proliferation

**Figure 2:**
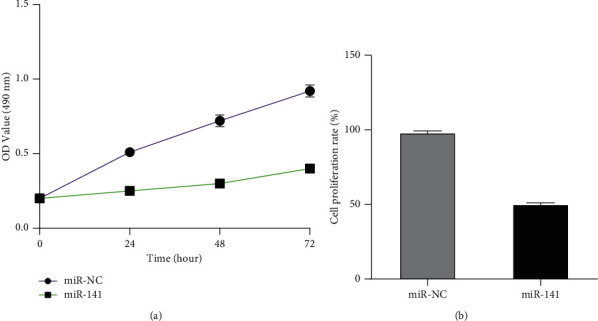
Effect of miR-141 overexpression on the proliferation of MHCC97H cells. (a) Cell proliferation curve detected by MTT. (b) Cell proliferation rate detected by MTT. Note: *P* < 0.05.

Transfect the already constructed overexpressed miR-141 vector plasmid into MHCC97H cells, and perform MTT assay. Through continuous measurement and analysis of 24 h, 48 h, and 72 h data, the cell growth and proliferation curve were obtained, to analyze the effect of miR-141 overexpression on liver cancer cell MHCC97H. Compared with the miR-NC in the negative control group, the cell proliferation was obviously inhibited by miR-141 overexpression (*P* < 0.05), as shown in Figure 2.

### 3.3. Effect of miR-141 Overexpression on Cell Migration and Invasion

In this study, the Transwell experiment was used to analyze the effect of miR-141 overexpression on the invasion and migration of liver cancer cells. The results showed that miR-141 overexpression had a significant impact on the invasion and migration ability of liver cancer cells, and the number of migrating cells decreased (*P* < 0.05) as shown in Table 1.

### 3.4. Effect of Low STAT4 Expression on Cell Proliferation

**Figure 3:**
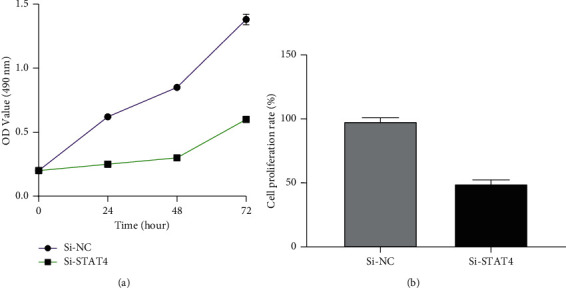
Effect of STAT4 interference on the proliferation of MHCC97H cells. (a) Cell proliferation curve detected by MTT. (b) Cell proliferation rate detected by MTT. Note: *P* < 0.05.

Transfection was carried out in the same way as above, and the MTT curve was drawn. The results showed that the cell proliferation rate of the Si-NC group was higher than that of the Si-STAT4 group (*P* < 0.05), as shown in Figure 3.

### 3.5. Effect of Low STAT4 Expression on Cell Migration and Invasion

In this study, the Transwell experiment was used to analyze the effect of low STAT4 expression on the invasion and migration of liver cancer cells. The results showed that the number of invaded and migrating cells in the Si-STAT4 group was less than that in the Si-NC group (*P* < 0.05), as shown in Table 2.

### 3.6. STAT4 Is the Downstream Target of miR-141 in Liver Cancer

**Figure 4:**
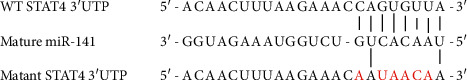
Schematic diagram of starBase prediction.

According to the previous test results, STAT4 is highly expressed in liver cancer tissues. Through bioinformatics Starbase, this study predicted that STAT4 can bind to miR-141, and the binding site and mutation sequence are shown in Figure 4. The above has evaluated the expression of STAT4 in liver cancer tissues and adjacent tissues, which was significantly upregulated (*P* < 0.05). The study further found that STAT4 was negatively correlated with miR-141 levels.

#### 3.6.1. Dual-Luciferase Reporter Experiment

**Figure 5:**
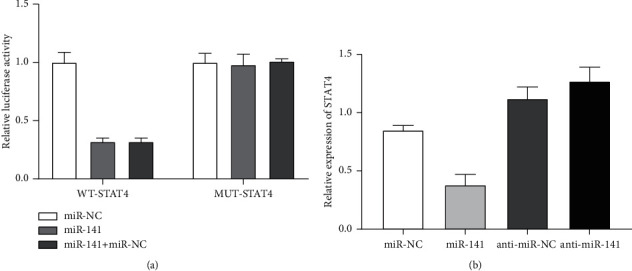
miR-141 targetedly regulates the expression of STAT4. (a) Luciferase activity of MHCC97H cells cotransfected by STAT4 reporter plasmid and miR-141. (b) Effect of miR-141 on STAT4 expression detected by Western blotting. *Note.* In (a), compared with the miR-NC group, *P* < 0.05; in (b), compared with the miR-NC group, *P* < 0.05, and compared with the anti-miR-NC group, *P* < 0.05.

The results of the dual-luciferase reporter experiment in this study showed that the luciferase activity did not change after the cotransfection of the mut-STAT4 group and miR-141 (*P* < 0.05), and the luciferase activity was significantly reduced after the cotransfection of the WT-STAT4 group and miR-141 group (*P* < 0.05), indicating that miR-141 can negatively regulate the expression of STAT4, as shown in Figure 5.

#### 3.6.2. MTT Experiment

**Figure 6:**
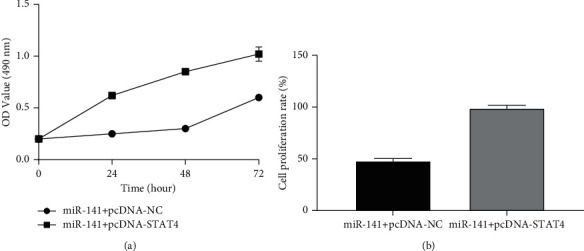
Overexpression of STAT4 can partially reverse the effect of miR-141 on MHCC97H cell proliferation. (a) Cell proliferation curve detected by MTT. (b) Cell proliferation rate detected by MTT. *Note*. *P* < 0.05.

Through the MTT experiment, it was found that miR-141 affected the proliferation of MHCC97H cells by regulating STAT4. The cell proliferation rate of the miR-141 + pcDNA-STAT4 group was higher than that of the miR-141+pcDNA-NC group (*P* < 0.05), as shown in Figure 6.

#### 3.6.3. Transwell Experiment

Through the Transwell experiment, it was found that the miR-141 + pcDNA-NC group had fewer migrating cells and invaded cells compared with the miR-141 + pcDNA-STAT4 group (*P* < 0.05), and the relative expression of Cyclin D1 protein in the miR-141 + pcDNA-NC group was also lower than that of the miR-141 + pcDNA-STAT4 group (*P* < 0.05). The results are shown in Table 3.

## 4. Discussion

MicroRNA (miRNA) is a small noncoding RNA that promotes its degradation or translational inhibition by binding to specific mRNA targets and regulates gene expression after transcription. The current studies have found that miRNAs play a role in regulating the physiological and pathological liver function. Changes in miRNA expression are associated with liver metabolic disorders, liver damage, liver fibrosis, and tumor development, which makes miRNA an attractive treatment strategy for the diagnosis and treatment of liver diseases. Based on most of the previous studies, miRNAs are often found in vulnerable parts related to tumors. Therefore, people began to study whether the abnormal expression of miRNAs would affect the occurrence and development of malignant tumors. Previous studies on miRNAs focused on a single miRNA, looking at its possible mechanisms in various cancers. As research goes deeper, scientists have gradually found that there are two or more miRNAs or miRNA families that participate in the biological process of cancer by targeting the same molecule. By consulting the literature, it has been found that miR-141 belongs to the miR-200a family, both of which are important members of the miRNAS family and are abnormally expressed in liver cancer. After comparison, it has been found that miR-200a and miR-141 have similar sequences, indicating that they have similar biological functions. This article takes miR-141 as the research object. Although studies have reported that low expression of miR-141 in liver cancer has an important antitumor effect, the mechanism of miR-141 in the process of tumorigenesis is still unclear yet.

Studies have found that the main reason for cancer metastasis is the gradual transfer and infiltration of cancer cells to surrounding tissues and blood vessels. Tumor cell migration refers to the directional movement of cells in the body, and tumor invasion refers to the penetration of tumor cells through the tissue barriers [11]. The above is also the reason why cancer patients cannot achieve good results after treatment [12]. After sorting out the current research data, it is found that STAT4 exists in different types of cancer and is a prognostic factor, which is consistent with the results obtained before. Through the detection of collected liver cancer tissues and cell lines, it is found that high STAT4 expression is associated with a poor prognosis, indicating that STAT4 plays an important role in promoting the generation and development of liver cancer and may be a potential oncogene. Based on the above findings, this study further analyzed the relationship between the expression level of miR-141 in liver cancer tissues and the clinicopathological characteristics of HCC patients. The results showed that the relative expression level of miR-141 was significantly related to the metastasis and cell invasion of liver cancer patients. miR-141 can improve the sensitivity and specificity of the diagnosis of liver cancer metastasis and invasion. These results indicate that miR-141 may play an important role in mediating the occurrence and development of liver cancer, and the detection of miR-141 has a potential clinical application value for early screening, diagnosis, and prognosis of liver cancer.

The main finding of this study is that miR-141 inhibits the proliferation and metastasis of liver cancer cells by targeting the expression of the STAT4 gene. Some reports have found that miR-141 can inhibit the proliferation of liver cancer cells by targeting sperm-associated antigen 9 (SPAG9), hepatocyte nuclear factor 3*β* (HNF 3*β*), zinc finger E-box (ZEB1), and so on. However, there are not many studies on the specific mechanism of miR-141 in liver cancer cells. This study proved through experiments and data that miR-141 can inhibit the proliferation and metastasis of liver cancer cells by targeting the gene STAT4, which provides some theoretical reference for the mechanism of miR-141 regulating STAT4 in liver cancer cells. As far as is known, this is the first study on miR-141 targeting the new gene STAT4 to regulate the proliferation and metastasis of liver cancer cells, and it has a certain application value.

To further clarify the problems in this study, we have successively constructed a series of stable liver cancer cell lines with overexpressed miR-141 and studied the biological function and mechanism of miR-141 at the cellular level. Cyclin D1 can positively regulate the cell cycle to promote cell proliferation. MMP2 and MMP9 are important inducers of cell migration and invasion. First, according to the western blot experiment, the expression of Cyclin D1, MMP2, and MMP9 decreased significantly, indicating that the overexpression of miR-141 can inhibit the proliferation, migration, and invasion of liver cancer cells. Secondly, after exploring the possible mechanism of this phenomenon, it is found that STAT4 is an important member of the STAT family and plays an important role in the transcription process, regulating the expression of multiple molecules, as well as in the cell proliferation and metastasis process. In the past ten years, scientists have proved that STAT4 single nucleotide polymorphism is closely related to the occurrence of liver cancer. Based on the above research, this article proved that the expression level of STAT4 is higher in liver cancer cells than in the normal cells and that, by inhibiting the expression of STAT4, the proliferation, migration, and invasion ability of liver cancer cells is greatly reduced. Finally, this study used bioinformatics software, luciferase reporter gene analysis, qRT-PCR, and western blotting to predict and confirm that STAT4 is the direct target gene of miR-141, and that, in liver cancer cells, miR-141 and STAT4 are negatively regulated, which means that miR-141 inhibits the growth and metastasis of liver cancer cell by targeting STAT4.

## 5. Conclusion

In summary, this study investigated the role of miR-141 in liver cancer cells from both clinical and cellular levels. The first step experiment shows that, compared with normal paracancerous tissues, the expression level of miRs in liver cancer tissues of liver cancer patients is apparently lower, indicating that the expression level of miR-141 is closely related to the clinicopathological characteristics of liver cancer, especially metastasis and invasion. This study reports a new target gene of miR-141-STAT4. At the cellular level, miR-141 reduces the expression of the proteins that promote the metastasis and proliferation of liver cancer by targeting STAT4 and coordinately regulates the proliferation, migration, and invasion of liver cancer cells. All of the above have greatly enriched the mechanism of the miR-141 family on liver cancer and provided a new experimental and theoretical basis for the application of miRNA in the early diagnosis, prognosis, and treatment of liver cancer.

## Figures and Tables

**Table 1:** Effects of miR-141 overexpression on the migration and invasion of MHCC97H cells *n* = 9, *x* ± *s*.

Group	Migrating cell no.	Invaded cell no.	miR-141	Cyclin D1	MMP2
mi-NC group	189 ± 19.27	169 ± 16.35	1.10 ± 0.23	1.32 ± 0.12	0.97 ± 0.10
miR-141 group	69 ± 15.56 ^*∗*^	60 ± 11.42 ^*∗*^	4.21 ± 0.22	0.49 ± 0.03	0.42 ± 0.03
T value	17.201	21.371	19.981	16.456	15.634
*P* value	0.05	0.05	0.05	0.05	0.05

*Note*. *n* = 9, *x* ± *s*. Compared with the miR-NC group, *P* < 0.05.

**Table 2:** Effects of low STAT4 expression on migration and invasion of MHCC97H cells.

Group	Migrating cell no.	Invaded cell no.	STAT4	Cyclin D1	MMP2
Si-NC group	225 ± 16.26	172.21 ± 15.03	1.20 ± 0.21	1.31 ± 0.15	0.96 ± 0.10
Si-STAT4 group	110 ± 10.46 ^*∗*^	85 ± 10.22 ^*∗*^	4.01 ± 0.32	0.47 ± 0.02	0.44 ± 0.01
*t* value	12.201	14.325	19.981	17.456	13.673
*P* value	0.05	0.05	0.05	0.05	0.05

*Note*. *n* = 9, *x* ± *s.* Compared with the Si-NC group, *P* < 0.05.

**Table 3:** Effects of miR-141 targetedly regulating STAT4 on cell migration and invasion.

Group	Migrating cell no.	Invaded cell no.	STAT4	Cyclin D1	MMP2
miR-141 + pcDNA-NC group	114 ± 11.23	72.24 ± 13.01	0.35 ± 0.02	0.51 ± 0.03	0.35 ± 0.02
miR-141 + pcNA-STAT4 group	192 ± 14.26	152.07 ± 12.12	0.82 ± 0.04	1.03 ± 0.10	0.86 ± 0.05

*t* value	12.401	14.564	15.781	13.453	15.543
*P* value	0.05	0.05	0.05	0.05	0.05

*Note*. *n* = 9, *x* ± *s*. Compared with miR-141 + pcDNA-NC group, *P* < 0.05.

## Data Availability

The datasets used and/or analyzed during the present study are available from the corresponding author on reasonable request.

## References

[1] Villanueva A. (2019). Hepatocellular carcinoma. *New England Journal of Medicine*.

[2] Marrero J. A., Kulik L. M., Sirlin C. B. (2018). Diagnosis, staging, and management of hepatocellular carcinoma: 2018 practice guidance by the American association for the study of liver diseases. *Hepatology*.

[3] Ye J., Wu S., Pan S., Huang J., Ge L. (2020). Risk scoring based on expression of long non-coding RNAs can effectively predict survival in hepatocellular carcinoma patients with or without fibrosis. *Oncology Reports*.

[4] Bracken C. P., Scott H. S., Goodall G. J. (2016). A network-biology perspective of microRNA function and dysfunction in cancer. *Nature Reviews Genetics*.

[5] Calin G. A., Sevignani C., Dumitru C. D. (2004). Human microRNA genes are frequently located at fragile sites and genomic regions involved in cancers. *Proceedings of the National Academy of Sciences*.

[6] Heusschen R., van Gink M., Griffioen A. W., Thijssen V. L. (2010). MicroRNAs in the tumor endothelium: novel controls on the angioregulatory switchboard. *Biochimica et Biophysica Acta*.

[7] Xu W. P., Liu J. P., Feng J. F. (2020). MiR-541 potentiates the response of human hepatocellular carcinoma to sorafenib treatment by inhibiting autophagy. *Gut*.

[8] Lin J., Shen J., Yue H., Cao Z. (2019). MiRNA-183-5p.1 promotes the migration and invasion of gastric cancer AGS cells by targeting TPM1. *Oncology Reports*.

[9] Zhang J., Xu S., Xu J. (2019). MiR-767-5p inhibits glioma proliferation and metastasis by targeting SUZ12. *Oncology Reports*.

[10] Velazquez-Torres G., Shoshan E., Ivan C. (2018). A-to-I miR-378a-3p editing can prevent melanoma progression via regulation of PARVA expression. *Nature Communications*.

[11] Khella H. W. Z., Bakhet M., Allo G. (2013). MiR-192, miR-194 and miR-215: a convergent microRNA network suppressing tumor progression in renal cell carcinoma. *Carcinogenesis*.

[12] Zhang W., Qian P., Zhang X. (2015). Autocrine/paracrine human growth hormone-stimulated MicroRNA 96-182-183 cluster promotes epithelial-mesenchymal transition and invasion in breast cancer. *Journal of Biological Chemistry*.

[13] Feng X., Wang Z., Fillmore R., Xi Y. (2014). MiR-200, a new star miRNA in human cancer. *Cancer Letters*.

[14] Belgardt B. F., Ahmed K., Spranger M. (2015). The microRNA-200 family regulates pancreatic beta cell survival in type 2 diabetes. *Nature Medicine*.

[15] Kim Y. K., Wee G., Park J. (2013). TALEN-based knockout library for human microRNAs. *Nature Structural & Molecular Biology*.

[16] Wang J., Song W., Shen W. (2017). MicroRNA-200a suppresses cell invasion and migration by directly targeting GAB1 in hepatocellular carcinoma. *Oncology Research Featuring Preclinical and Clinical Cancer Therapeutics*.

[17] Feng J., Wang J., Chen M. (2015). MiR-200a suppresses cell growth and migration by targeting MACC1 and predicts prognosis in hepatocellular carcinoma. *Oncology Reports*.

[18] Hou X., Yang L., Jiang X. (2019). Role of microRNA-141-3p in the progression and metastasis of hepatocellular carcinoma cell. *International Journal of Biological Macromolecules*.

[19] Zhao Y., Xu Z., Zhou J., Yang H. (2019). MiR-141 inhibits proliferation, migration and invasion in human hepatocellular carcinoma cells by directly downregulating TGF*β*R1. *Oncology Reports*.

[20] Mansini A. P., Lorenzo Pisarello M. J., Thelen K. M. (2018). MicroRNA (miR)-433 and miR-22 dysregulations induce histone-deacetylase-6 overexpression and ciliary loss in cholangiocarcinoma. *Hepatology*.

[21] Lou G., Dong X., Xia C. (2016). Direct targeting sperm-associated antigen 9 by miR-141 influences hepatocellular carcinoma cell growth and metastasis via JNK pathway. *Journal of Experimental & Clinical Cancer Research*.

[22] Zheng L., Xu M., Xu J. (2018). ELF3 promotes epithelial-mesenchymal transition by protecting ZEB1 from miR-141-3p-mediated silencing in hepatocellular carcinoma. *Cell Death & Disease*.

[23] Wu L., Pan C., Wei X. (2018). lncRNA KRAL reverses 5-fluorouracil resistance in hepatocellular carcinoma cells by acting as a ceRNA against miR-141. *Cell Communication and Signaling*.

[24] Lin L., Liang H., Wang Y. (2014). MicroRNA-141 inhibits cell proliferation and invasion and promotes apoptosis by targeting hepatocyte nuclear factor-3*β* in hepatocellular carcinoma cells. *BMC Cancer*.

